# Comparison of Latanoprost/Timolol with Carbonic Anhydrase Inhibitor and Dorzolamide/Timolol with Prostaglandin Analog in the Treatment of Glaucoma

**DOI:** 10.1155/2014/975429

**Published:** 2014-03-05

**Authors:** Kenji Inoue, Shoichi Soeda, Goji Tomita

**Affiliations:** ^1^Inouye Eye Hospital, 4-3 Kanda-Surugadai, Chiyoda-ku, Tokyo 101-0062, Japan; ^2^Nishikasai Inouye Eye Hospital, Tokyo 134-0088, Japan; ^3^Department of Ophthalmology, Toho University Ohashi Medical Center, Tokyo 153-8515, Japan

## Abstract

*Purpose.* We retrospectively reviewed medical records of glaucoma patients to investigate how switching medications may affect intraocular pressure (IOP) management. Three concomitant medications were changed to two medications: one combination drop and one single-action drop. Associated adverse effects were also examined. *Subjects and Methods.* A total of 112 patients with primary open-angle glaucoma or ocular hypertension were examined. All patients were concomitantly using a prostaglandin (PG) analog, a **β**-blocker, and a carbonic anhydrate inhibitor (CAI). Fifty-five patients began using latanoprost (PG analog)/timolol (**β**-blocker) fixed-combination (LTFC) drops and a CAI (group 1), and 57 patients began using dorzolamide (CAI)/timolol fixed-combination (DTFC) drops and a PG analog (group 2). The IOP was measured every 6 months for 2 years following medication changes. Changes in visual field mean deviation (MD) and medication discontinuations were also examined. *Results.* There were no significant differences in IOP or MD values before and after medication changes in either group. The proportion of medication discontinuations, uncontrolled IOP, and adverse reactions was similar in both groups. *Conclusion.* Switching patients from multiple single-action medications to combination medications was not associated with changes in IOP, visual field testing results, or adverse event frequency.

## 1. Introduction

Fixed-combination eye drops for treating glaucoma were developed to improve patient compliance. 0.005% latanoprost + 0.5% timolol maleate fixed-combination (LTFC) eye drops, 0.005% travoprost + 0.5% timolol fixed-combination eye drops, and 1% dorzolamide + 0.5% timolol maleate fixed-combination (DTFC) eye drops became available in Japan three years ago.

When a single medication does not achieve sufficient reductions in intraocular pressure (IOP), additional eye drops are often added to the patient's medication regimen. Because of their efficacy in decreasing IOP and the low frequency of adverse reactions, topical prostaglandin (PG) analogs, *β*-blockers, and carbon anhydrase inhibitors (CAI) are most often used. Combining 2 drugs into one fixed-combination eye drop decreases the number of eye drops patients must administer and the frequency in which they must be used, both of which improve medication compliance. Some physicians choose to switch patients from a three-drug regimen to a two-drop regimen by switching PG analogs and *β*-blockers to prostaglandin/timolol fixed-combination eye drops or from *β*-blockers and CAIs to DTFC eye drops. Unfortunately, only one previous report has investigated the impacts of such medication changes [1].

Here, we retrospectively investigated changes in IOP, effects on the visual field, and incidences of adverse effects in patients moving from a three-drop medication regimen to a two-drop medication regimen through the use of fixed-combination drugs. All patients were initially using PG analogs, *β*-blockers, and CAIs and were switched to either LTFC with CAI or DTFC with PG analog eye drops. All patients included in the study were followed for 2 years.

## 2. Subjects and Methods

We retrospectively investigated 112 eyes from 112 patients (57 men and 55 women) diagnosed with primary open-angle glaucoma or ocular hypertension at Inouye Eye Hospital or Nishikasai Inouye Eye Hospital between April 2010 and February 2011. Patients were concomitantly using 3 types of eye drops to control IOP, which included a PG analog, a *β*-blocker, and a CAI. The PG analogs and *β*-blockers were replaced with LTFC eye drops (group 1) and *β*-blockers and CAI were replaced with DTFC eye drops (group 2).

Patients were excluded from analyses if they had undergone previous glaucoma surgery, had cataract surgery within 3 months, were using corticosteroid eye drops, or had corneal disease that could influence tonometry readings. At the discretion of the patient and/or their treating physician, combination medications were discontinued when an adverse reaction occurred; when IOP was too high and was accompanied by a medication change; or when selective laser trabeculoplasty, cataract, or glaucoma surgery was required. If both eyes met inclusion criteria, the eye with the higher IOP was selected for analyses. If both eyes had the same IOP, then the right eye was selected to use in analyses.

Patient characteristics for groups 1 and 2 are shown in Table 1. In both groups, the drug regimen change was not preceded by a washout period. Additionally, treatment with the third drop continued unchanged (CAI in group 1; PG analogs in group 2). The IOP was measured 6, 12, 18, and 24 months after the regimen switch with a Goldmann applanation tonometer. All measurements were performed by the same examiner at nearly the same time of day. The IOP measurements were compared to those obtained prior to the change in drug regimen (ANOVA or Bonferroni/Dunnett test). A Humphrey visual field test (30–2 SITA-Standard program) was used to evaluate visual field function prior to changing to the fixed-combination eye drop and repeated 12 and 24 months after the change. Mean deviation (MD) values were compared (Friedmann test) between baseline and after the medication switch. The presence of adverse reactions was investigated at every follow-up visit. Statistical significance was defined as *P* < 0.05.

## 3. Results

**Figure 1:**
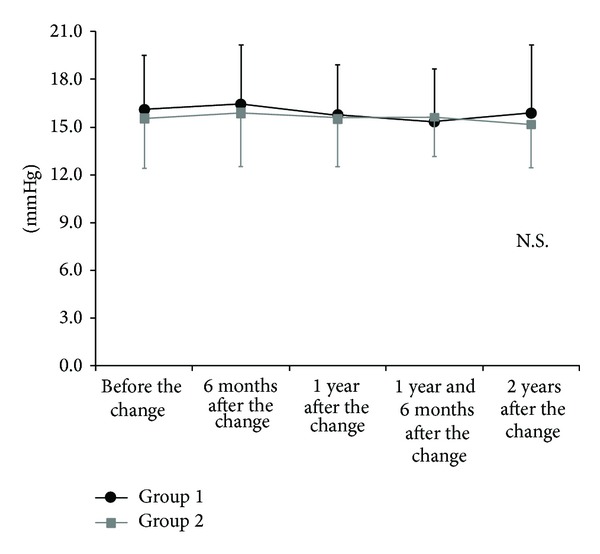
Intraocular pressure (IOP) before and after changing to latanoprost/timolol maleate fixed-combination eye drops or to dorzolamide/timolol maleate fixed-combination eye drops (ANOVA or Bonferroni/Dunnett analysis). The IOP was not significantly different from baseline at any time point examined in either medication group. Average data are presented and error bars represent one standard deviation.

There were no significant differences between groups in the ratio of men to women, age, or disease type. Additionally, no differences were observed in IOP or the MD value before the medication change (Table 1). In group 1, IOP was 16.4 ± 3.7 mmHg (average ± standard deviation) 6 months after the change, 15.7 ± 3.1 mmHg 12 months after the change, 15.4 ± 3.3 mmHg 18 months after the change, and 15.9 ± 4.3 mmHg 24 months after the change. Each of these IOP measurements was not statistically different from that obtained before the change (16.1 ± 3.4 mmHg; *P* = 0.47; Figure 1). In group 2, IOP was 15.9 ± 3.3 mmHg 6 months after the change, 15.6 ± 3.1 mmHg 12 months after the change, 15.6 ± 2.4 mmHg 18 months after the change, and 15.1 ± 2.7 mmHg 24 months after the change. As in group 1, these values were not statistically different from that obtained before the change (15.6 ± 3.2 mmHg; *P* = 0.29).

**Figure 2:**
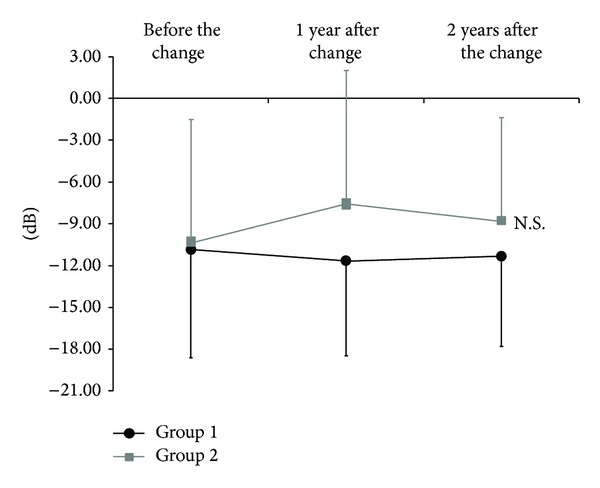
Mean deviation values before and after changing to latanoprost/timolol maleate fixed-combination eye drops or to dorzolamide/timolol maleate fixed-combination eye drops (Friedmann analysis). There were no significant differences from baseline in mean deviation values in either medication group at any time point examined. Average data are presented and error bars represent one standard deviation.

Visual field testing results for group 1 yielded an MD value of −10.85 ± 8.81 dB before the change, −11.6 ± 9.67 dB 12 months after the change, and −11.31 ± 7.39 dB 24 months after the change. There was no significant difference among the time points examined (*P* = 0.06; Figure 2). In group 2, the MD values were −10.32 ± 7.77 dB before the change, −7.64 ± 6.84 dB 12 months after the change, and −8.78 ± 6.46 dB 24 months after the change. There was no significant difference among time points examined (*P* = 0.50).

Four out of 55 patients (7.3%) in group 1 and 8 out of 57 cases (14.0%) in group 2 discontinued fixed-combination medications because of adverse reactions (Table 2). Additionally 11 out of 55 patients (20.0%) in group 1 and 14 out of 57 patients (24.6%) in group 2 discontinued fixed-combination medications because of elevated IOP or the need for glaucoma surgery. The slight differences in discontinuation because of adverse events (*P* = 0.36) and elevated IOP/glaucoma surgery (*P* = 0.65) were not statistically significant.

## 4. Discussion

Few reports have examined the effects of switching from a 3-drug regimen to a 2-drop regimen (one single medication eye drop and one fixed-combination eye drop). Nakakura et al. [1] investigated this in patients with primary open-angle glaucoma who were using PG analogs, *β*-blockers, and CAI concomitantly. In that study, each patient was switched to either LTFC with brinzolamide eye drops (20 cases) or to DTFC with latanoprost eye drops (16 cases). Four and 12 weeks after the change, no significant change from baseline was observed in IOP and the risk of developing a corneal epithelial disorder or conjunctival injection had not significantly changed. In the present study, there were no significant differences in IOP before and after a similar medication change. However, about 20% of patients in each group had an increase in IOP and were discontinued from the new medication regimen. When LTFC eye drops were used, the administration frequency of timolol decreased from 2 times a day to once a day. When DTFC eye drops were used, the administration frequency of dorzolamide decreased from 3 times a day to 2 times a day. Increases in IOP were often observed in patients who had satisfactory medication compliance. In contrast, when patients with poor compliance on the 3-drop regimen changed to the 2-drop regimen (both administration frequency and number of medication bottles decreased), IOP also generally decreased. There was no change in average IOP, when patients who were not compliant before the medication changed were added into the analyses.

Many reports have been published on changing PG analogs and *β*-blockers to LTFC eye drops and changing CAI and *β*-blockers to DTFC eye drops [2–11]. The LTFC eye drops had varying effects on IOP [2–7], but IOP did not change after switching to DTFC eye drops [8–11]. Our results are in agreement with these observations.

The visual field was preserved for at least 1 year following medication changes to LTFC [3] and DTFC [8], as determined by MD values. In agreement with previous studies, we found no significant differences from baseline in MD values obtained 1 and 2 years after the medication switch. Therefore, we believe that fixed-combination eye drops somehow contribute to long-term visual field preservation.

Adverse reactions associated with the use of LTFC eye drops include hyperemia, feeling of stimulation, itching, photophobia, foreign body sensation, superficial punctate keratitis, conjunctivitis, corneal epithelial disorder, and headache [1–7]. Adverse reactions associated with the use of the DTFC eye drops include hyperemia, feelings of stimulation, itching, foreign body sensations, conjunctivitis, superficial punctate keratitis, headache, bitter taste, and blurred vision [1, 8–11]. We observed similar adverse reactions in the current study, which included blurred vision, feeling of stimulation, chest pain, and asthma attack. Chest pain and asthma have been associated with the timolol maleate contained in the DTFC eye drops.

Our patients had primary open-angle glaucoma or ocular hypertension and were using PG analogs, *β*-blockers, and CAIs. They were changed to either LTFC or DTFC eye drops, after which IOP and visual field results remained stable for 2 years. No serious adverse reactions were observed in any patient. However, IOP increased in approximately 20% of cases in both groups.

## Figures and Tables

**Table:** (a)

	Group 1	Group 2	*P*
Cases	55 cases	57 cases	—

Ratio of male and female	31 : 24	26 : 31	0.2648

Age	66.2 ± 12.4 years old	67.1 ± 12.4 years old	0.7161
	32~85 years old	27~85 years old	

Disease type			
Primary open-angle glaucoma	40 cases	44 cases	0.8194
Normal tension glaucoma	12 cases	11 cases	
Ocular hypertension	3 cases	2 cases	

IOP before the change	16.1 ± 3.4 mmHg	15.6 ± 3.2 mmHg	0.367
	10~24 mmHg	10~23 mmHg	

MD value before the change	−10.85 ± 8.81 dB	−10.32 ± 7.77 dB	0.8772
	−33.15~−0.13 dB	−21.73~−0.09 dB	

**Table:** (b)

	Used eye drops	Group 1	Group 2	*P*
		55 cases	57 cases	—
Before the change	PG analogs			
	Latanoprost	52	44	0.0139
	Travoprost	—	7	
	Tafluprost	3	3	
	Bimatoprost	—	3	
	*β*-blocker			
	Timolol	51	39	0.0016
	Carteolol	4	18	
	CAI			
	Dorzolamide	13	38	<0.0001
	Brinzolamide	42	19	

After the change	PG analogs			
	Latanoprost	—	44	—
	Travoprost	—	7	
	Tafluprost	—	3	
	Bimatoprost	—	3	
	*β*-blocker			
	Timolol	—	—	—
	Carteolol	—	—	
	CAI			
	Dorzolamide	13	—	—
	Brinzolamide	42	—	
	Fixed-combination eye drops			
	Latanoprost/timolol	55	—	—
	Dorzolamide/timolol	—	57	—

**Table 2:** Cases discontinued after the switch to fixed-combination eye drops.

	Group 1	Group 2	*P*
IOP decreasing efficacy Glaucoma surgery conducted	20.0% (11 cases)	24.6% (14 cases)	0.6522

Adverse reactions	7.3% (4 cases)	14.0% (8 cases)	0.3612

Classification of adverse reactions	Eye irritation 1 case	Blurred vision 3 cases	
	Itchiness 1 case	Eye irritation 3 cases	
	Foreign body sensation 1 case	Chest pain 1 case	
	Superficial punctate keratopathy 1 case	Asthma attack 1 case	

## References

[1] Nakakura S, Tabuchi H, Baba Y (2012). Comparison of the latanoprost 0.005%/timolol 0.5% + brinzolamide 1% versus dorzolamide 1%/timolol 0.5% + latanoprost 0.005%: a 12-week, randomized open-label trial. *Clinical Ophthalmology*.

[2] Diestelhorst M, Larsson L-I (2004). A 12 week study comparing the fixed combination of latanoprost and timolol with the concomitant use of the individual components in patients with open angle glaucoma and ocular hypertension. *British Journal of Ophthalmology*.

[3] Inoue K, Okayama R, Higa R, Wakakura M, Tomita G (2012). Assessment of ocular hypotensive effect and safety 12 months after changing from an unfixed combination to latanoprost 0.005% + timolol maleate 0.5% fixed combination. *Clinical Ophthalmology*.

[4] Kiuchi T, Inoue T, Tachibayashi N, Oshika T (2012). Long-term efficacy after switching from unfixed combination of latanoprost and timolol maleate to fixed combination. *Journal of the Eye*.

[5] Polo V, Larrosa JM, Ferreras A, Borque E, Pablo LE, Honrubia FM (2008). Effect on diurnal intraocular pressure of the fixed combination of latanoprost 0.005% and timolol 0.5% administered in the evening in glaucoma. *Annals of Ophthalmology*.

[6] Dunker S, Schmucker A, Maier H (2007). Tolerability, quality of life, and persistency of use in patients with glaucoma who are switched to the fixed combination of latanoprost and timolol. *Advances in Therapy*.

[7] Hamacher T, Schinzel M, Schölzel-Klatt A (2004). Short term efficacy and safety in glaucoma patients changed to the latanoprost 0.005%/timolol maleate 0.5% fixed combination from monotherapies and adjunctive therapies. *British Journal of Ophthalmology*.

[8] Inoue K, Tomita G (2013). Twelve-month evaluation of dorzolamide hydrochloride 1%/timolol maleate 0.5% fixed combination eye drops after switch from unfixed combination. *Journal of the Eye*.

[9] Shimamura S, Ohashi H, Kawai K (2012). Comparison of the efficacy and safety of the dorzolamide-timolol combination in non-adherent patients who had been prescribed glaucoma medication. *Folia Ophthalmologica Japonica*.

[10] Inoue K, Shiokawa M, Sugahara M (2012). Three-month evaluation of ocular hypotensive effect and safety of dorzolamide hydrochloride 1%/timolol maleate 0.5% fixed combination drops after discontinuation of carbonic anhydrase inhibitor and *β*-blockers. *Japanese Journal of Ophthalmology*.

[11] Takeda S, Uemura A, Matsubara M (2012). Efficacy and safety of switch to the dorzolamide/timolol fixed combination in glaucoma patients. *Journal of the Eye*.

